# Positive & Negative Roles of Innate Effector Cells in Controlling Cancer Progression

**DOI:** 10.3389/fimmu.2018.01990

**Published:** 2018-09-21

**Authors:** Dorian Stolk, Hans J. van der Vliet, Tanja D. de Gruijl, Yvette van Kooyk, Mark A. Exley

**Affiliations:** ^1^Department of Molecular Cell Biology and Immunology, VU University Medical Center, Amsterdam, Netherlands; ^2^Department of Medical Oncology, VU University Medical Center, Amsterdam, Netherlands; ^3^Faculty of Biology, Medicine and Health, University of Manchester, Manchester, United Kingdom; ^4^Harvard Medical School, Brigham and Women's Hospital, Boston, MA, United States; ^5^Agenus, Inc., Lexington, MA, United States

**Keywords:** NKT, iNKT cells, CD1d, MAIT cells, gamma-delta T cells, NK cells, cancer immunotherapy

## Abstract

Innate immune cells are active at the front line of host defense against pathogens and now appear to play a range of roles under non-infectious conditions as well, most notably in cancer. Establishing the balance of innate immune responses is critical for the “flavor” of these responses and subsequent adaptive immunity and can be either “good or bad” in controlling cancer progression. The importance of innate NK cells in tumor immune responses has already been extensively studied over the last few decades, but more recently several relatively mono- or oligo-clonal [i.e., (semi-) invariant] innate T cell subsets received substantial interest in tumor immunology including invariant natural killer T (iNKT), γδ-T and mucosal associated invariant T (MAIT) cells. These subsets produce high levels of various pro- and/or anti-inflammatory cytokines/chemokines reflecting their capacity to suppress or stimulate immune responses. Survival of patients with cancer has been linked to the frequencies and activation status of NK, iNKT, and γδ-T cells. It has become clear that NK, iNKT, γδ-T as well as MAIT cells all have physiological roles in anti-tumor responses, which emphasize their possible relevance for tumor immunotherapy. A variety of clinical trials has focused on manipulating NK, iNKT, and γδ-T cell functions as a cancer immunotherapeutic approach demonstrating their safety and potential for achieving beneficial therapeutic effects, while the exploration of MAIT cell related therapies is still in its infancy. Current issues limiting the full therapeutic potential of these innate cell subsets appear to be related to defects and suppressive properties of these subsets that, with the right stimulus, might be reversed. In general, how innate lymphocytes are activated appears to control their subsequent abilities and consequent impact on adaptive immunity. Controlling these potent regulators and mediators of the immune system should enable their protective roles to dominate and their deleterious potential (in the specific context of cancer) to be mitigated.

## Introduction

The importance of the immune system in tumor control and development has been extensively studied and it has been shown that different elements of the innate and adaptive immune system can exhibit anti-tumor activity. Adaptive immune cells are antigen-specific and have enhanced responses to subsequent antigen exposure. Innate-like or semi-invariant T cell subsets can recruit adaptive responses and thereby support eradication of tumor cells. It has become more and more apparent that besides conventional B and T cells and classical NK lymphocytes, other conserved innate T cells, such as natural killer T cells (NKT), γδ T cells and mucosa associated invariant (MAIT) cells, are of great importance in controlling tumor growth. Compared with conventional T cells, these innate T cell subsets are characterized by a limited (γδ T cells) or even (semi)-invariant (iNKT cell populations and MAIT cells) T cell receptor (TCR) repertoire and can have a dual role in tumor immunity. On one hand, they can stimulate or even directly mediate anti-tumor responses, but on the other hand their regulatory functions may hamper tumor eradication. A deeper understanding of the roles of classical NK cells and these innate T cell subsets in tumor immune biology, has led to new therapeutic options for cancer, whereby manipulation of these invariant subsets has already shown early signs of promising anti-tumor efficacy.

In this Review, we will briefly introduce and then outline our current understanding of the functions and potential of the classical innate NK cells and several semi-invariant subsets of innate immune T cells, and highlight their role in controlling anti-tumor immune responses as well as their therapeutic potential.

## Innate lymphocyte subsets in natural and therapeutic anti-tumor immunity

### NK cells

**Figure 1:**
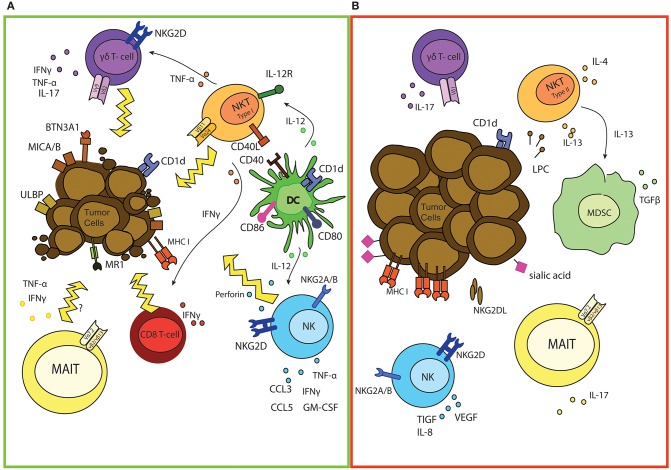
The good and bad of (semi invariant) innate cells in cancer. **(A)** Overview of anti-tumor responses of NK, iNKT, γδ, and MAIT cells. Activated iNKT can directly kill tumor cells and promote DC triggering which is marked by up regulation of co-stimulatory molecules and enhanced cross-presentation capacities of DCs. iNKT can also directly promote effector T cell activation and differentiation and stimulate γδ mediated anti-tumor responses by secretion of different cytokines. Indirectly, iNKT also support activation of NK cells via IL-12 release of DCs, thereby enhancing anti-tumor effector functions. Expression of MICA/B and ULBP proteins on tumor cells induces activation of both γδ T and NK cells. As a result γδ T cells and NK release different pro-inflammatory cytokines for immune support and are also capable of directly killing malignant cells. Loss of expression of MHC-I molecules serves as another NK activating trigger, leading to perforin release and tumor cell eradication. As well as NK, iNKT, and γδ T cells, tumor infiltrating MAIT cells could also secrete different pro-inflammatory cytokines and potentially kill cancerous cells. **(B)** Potential tumor-promoting functions of NK, iNKT, γδ, and MAIT cells. Tumor cells possess different mechanisms to escape/manipulate NK cells, leaving NK unable to lyse malignant cells. NK also secrete immune suppressive and angiogenesis stimulating cytokines, which promote tumor growth. As well as functional defects of iNKT, which are marked by decreased IFNγ/IL-4 ratio, cancer cells can also skew iNKT function via secretion of lysophosphatidylcholine (LPC), resulting in IL-13 production by iNKT. This induces production of immuno-suppressive cytokines by MDSC. Also release of IL-17 can promote tumor growth. γδ and MAIT cells and a minor population of NKT cells can also release IL-17, which can inhibit Th1-type responses.

Natural killer cells (NK) comprise a classical innate lymphoid cell subset that plays an important role in the defense against infections and cancer (1). NK possess potent cytolytic activity to rapidly kill targeted cells (e.g., virally infected or malignant) and secrete various effector cytokines and chemokines like IFNγ, TNFα, GM-CSF, MIP-1α (CCL3), and RANTES (CCL5) (2, 3) (Figure 1). Because of this variety in secreted cytokines, NK activity is also important for proper function of other innate immune subsets such as DCs and macrophages (4, 5). But also in adaptive immune responses, such as cytokine secretion of T and B cells, NK cells seem to have an important contribution (6–9) and therefore NK activation can support tumor specific immune responses.

NK activation is based on the balance between inhibitory and activating signals from various receptors based on “missing-self” and “induced-self” ligand interactions. Most important activating receptors are the natural cytotoxicity receptors (NCRs) NKp46, NKp30, NKp44, the C-type lectin NKG2D, the FcR CD16 and some killer cell immunoglobulin-like receptors (KIRs), while the inhibitory receptors include CD94/NKG2A/B and KIR-2DL and KIR-3DL (10). The feature of “missing-self” recognition is based on the situation in which expression of NK-inhibitory MHC-I molecules in the steady state dominates over the expression of NK activating ligands, thereby leaving NK inactive. In contrast, the increased expression of “induced-self” ligands on malignant cells in combination with reduced levels of MHC-I, leads to strong NK triggering and the induction of potent cytolytic activity.

The finding that MHC-I deficient syngeneic tumors were selectively rejected by NK and that the detection of the absence of MHC-I was mediated via inhibitory receptors on NK (10–13) has led to the discovery of multiple indications in which NK are involved in tumor eradication. Different mouse studies using transplanted syngeneic tumor cells showed that either genetic or antibody mediated NK depletion led to increased tumor growth and higher metastasis rates (14–17). Tumor outgrowth could be inhibited through addition of various cytokines that enhance NK activity. Models using methylcholanthrene (MCA) for chemical induction of tumors in combination with NK depletion demonstrated a role for NK, much like iNKT, in immune surveillance at early stages of tumor development (18). Mice deficient for important NK effector molecules such as perforin, IFNγ and the downstream signaling molecule of the IFNγ receptor, STAT1, developed tumors in higher frequencies than WT mice (1, 19). More sophisticated models using RAG2/γc deficient mice, which lack all lymphocytes including NK, iNKT, γδ T, classical CD4^+^ and CD8^+^ αβ T cells and B cells, showed a higher incidence of tumor growth compared to RAG2 deficient mice alone (which lack αβ T cells and B cells) demonstrating that indeed NK cells are in part responsible for inhibiting tumor growth (20). However, caution is called for the interpretation of these data since these models did not exclusively eliminate NK.

Also in human tumors correlative analyses have indicated a role for NK in tumor elimination. In cancer patients different NK deficiencies and dysfunctionalities have been observed (1, 2, 21–24) and an 11 year follow-up study highlighted that NK function can be a good indicator for cancer development and progression (25). In addition, multiple groups have reported that high levels of tumor infiltrating NK cells (TINKs) represents a favorable outcome for patients with different types of carcinomas and therefore NK cell infiltration appears to be a positive prognostic marker which may also respond to IL-12 (26–28).

Tumors can develop different strategies to evade NK mediated lysis (29) (Figure 1B). For example in acute myeloid leukemia, leukemic cells could induce loss or decrease of NCR expression on NK (30, 31) and this phenotype was correlated with a decreased killing capacity (31). Another mechanism by which tumors evade immune surveillance by NK is upregulation of classical and non-classical MHC-I molecules that reduce NK activity by delivery of inhibitory signals (32–34) (Figure 1B). Also specific manipulation of NKG2D signaling has been observed in tumors and can explain why in some cases the presence of NKG2D ligands is not sufficient for tumor clearance, but rather promotes tumor growth through NK cell immune subversion. Normally expression of NKG2D ligands such as the MHC class I chain-related molecules (MIC) A/B and members of the UL-16 binding protein (ULBP) family leads to activation on NK and in patients with colorectal carcinoma expression of MICA even correlates with good prognosis (35). However, tumor cells can release soluble forms of NKG2D ligands and elevated levels of MICA/B and ULBP2 have been detected in sera of patients with various epithelial and hematopoietic malignancies (36–41) (Figure 1B). Soluble NKG2D ligands can abrogate NK activation and down regulate and block NKG2D on tumor infiltrating lymphocytes (37, 38, 42). Recently it has become clear that also aberrant glycosylation on tumor cells affects NK activity. Jandus et al. demonstrated that sialic acid containing carbohydrates on tumor cells serve as ligands for the siglec 7/9 receptors on NK and interfere with NK mediated anti-tumor responses (43). This hypothesis is supported by findings that enzymatic induction of high sialylation on tumors dampens activity of NK (44) (Figure 1B).

Different studies therefore indicate that NK functions can be turned off in the presence of a tumor, but the coin doesn't always flip from “good” to “inactive” but rather flips to “bad”, leaving NK pro-tumorigenic. This has e.g., been described by Bruno et al. who identified NK cells in patients with non-small cell lung cancer that produced substantial levels of vascular endothelial growth factor (VEGF), placental growth factor (PIGF) and IL-8 and therefore might stimulate angiogenesis to enhance tumor growth (45, 46) and actively suppress immune responses (47) (Figure 1B).

Therapeutically, it is a challenge to overcome the escape mechanisms that tumors have developed to prevent NK killing and to reverse NK paralysis. Many different strategies are currently tested and some show promising results in preclinical and increasingly clinical studies (10). Most studies have focused on adoptive transfer of autologous, allogeneic or NK cell lines to enhance NK cytotoxicity against tumors. All three approaches show anti-tumor activity but with various efficacy (48–53). Indeed, allogeneic hemopoietic stem cell transplant anti-leukemia effects are partly mediated by NK cells (49–53). Other promising approaches include blockade of inhibitory receptors on NK using mAb which could recover effective NK mediated killing activity (54–56) and chimeric antigen receptor (CAR) technology that after extensive testing in T cells, have also been applied to NK cells and might constitute a promising approach to enhance NK cell mediated anti-tumor responses (51–53, 57–59). Indeed, autologous CAR-NK might be one way to avoid issues of contaminating allogeneic T cells whilst augmenting the NK activity specifically against the tumor, where appropriate CAR targets are available.

To conclude, the relevance of NK in tumor immune responses has been revealed in many studies. However, immune editing of the tumor and immune suppression perpetrated by the tumor can abrogate NK function limiting NK mediated lysis of tumor cells. More insight in the exact contribution of NK cells in tumor progression and ways to overcome NK paralysis is warranted to optimize NK activating therapies.

### NKT cell populations

There are 2 major populations of CD1d-restricted “NKT” cells (T cells sharing some NK phenotypic and functional properties): The better-known “Invariant natural killer T cells” (iNKT) and polyclonal diverse “non-invariant” NKT cells (60, 61). iNKT are a subset of lymphocytes with a significant role in regulating immune responses, including immune surveillance against tumors. iNKT recognize lipid antigens presented by the monomorphic MHC-like molecule CD1d, predominantly expressed by dendritic cells (DC) and other antigen presenting cells (APC). iNKT were initially identified by their restricted TCR repertoire (Vα14Jα18 in mice and Vα24Jα18 in humans), but subsets expressing variable TCRs do also exist. The basis of the regulatory function of iNKT appears to lie in their capacity for rapid secretion of multiple cytokines upon TCR triggering which is accompanied by an increased CD1d-restricted cytotoxic capacity (60). Cytokines released by iNKT include both regulatory cytokines (e.g., IL-4, IL-10, IL-13) as well as pro-inflammatory cytokines such as IL-2, IL-17, and IFNγ, reflecting their capacity to suppress or stimulate immune responses (61, 62). iNKT cell cytotoxic activity can be mediated by classical granule-mediated mechanisms, although Fas / FasL dependent killing has also be reported (60, 61) (Figure 1A).

iNKT recognize different microbial and endogenous antigens such as gangliosides and glycolipids and therefore play a substantial role during infection (63). However the compound most efficient for activating iNKT is the marine sponge derived glycolipid α-galactosylceramide (αGalCer). Ever since the identification of αGalCer as prototypic high-affinity CD1d binding lipid and potent iNKT stimulant, studies have shown that iNKT activation with αGalCer promotes tumor rejection and protects from the development of metastases in multiple murine tumor models (64–67). This anti-tumor effect could be further improved by injection of αGalCer-pulsed DCs and anti-metastatic effects were shown to be driven by IFNγ (68–70). Furthermore, IL-12, a master regulator of Th1 responses, like αGalCer, drives the anti-metastatic activity of T cells, including iNKT, as well as NK cells and effects of low dose IL-12 treatment in murine tumor models can be predominantly mediated by the activity of iNKT (66, 71–74).

Whereas multiple studies have shown the critical role of iNKT in the induction of potent anti-tumor responses in response to stimulation by the above-mentioned exogenous factors such as αGalCer and IL-12, the physiological role of these cells in tumor immunity remains more elusive. However, Smyth et al indicated that, at least in a model of MCA-induced fibrosarcomas, iNKT fulfill an essential role in tumor immune surveillance. Adoptive transfer of iNKT from wild type mice into iNKT cell deficient mice (Jα18 –/–) clearly showed a protective effect on tumor outgrowth without a requirement of additional exogenous stimuli (75). The contribution of iNKT cells to immune surveillance has also been highlighted by findings on their capacity to mature DC and subsequently activate NK and cytotoxic CD8^+^ T cells, the latter two of which then become potent cytotoxic cells. Upon recognition of CD1d:lipid complexes and the costimulatory molecules CD80/86 on the surface of DCs, iNKT cells up-regulate the IL-12R (66, 71–74) and CD40L molecule. Subsequently, and mediated by CD40L, iNKT induce DC maturation and release of IL-12. This IL-12 release in turn potently increases IFNγ production by iNKT which then, together with enhanced cross-presentation of DCs after iNKT induced maturation, boosts activation of anti-tumorigenic cytotoxic T lymphocytes (CTL) (76, 77) (Figure 1A). In other words, iNKT have the capacity to jump-start immune responses and together with DCs to bridge the innate and adaptive immune systems.

Besides providing a pro-inflammatory status by interaction with DCs, NK and CTL, iNKT have also been found to be able to control tumor growth by killing tumor supportive IL-6-producing CD1d^+^ CD68^+^ tumor associated macrophages (TAM) (78). Moreover, iNKT could potentially also control myeloid derived suppressor cells (MDSC) in the tumor microenvironment (TME) (79, 80). Absence of iNKT in mice infected with influenza virus resulted in strong expansion of MDSC, but interestingly adoptive transfer of iNKT could abolish suppressive activity of MDSCs. So, by targeting TAM and MDSC, iNKT may skew the TME to a pro-immune milieu.

While the function of iNKT as regulators of immune responses has been widely acknowledged (81, 82), the exact mechanisms polarizing iNKT effector functions remain elusive, thusfar in part limiting their therapeutic potential in clinical trials. Studies in multiple human cancers have revealed selective numerical and/or functional defects in the iNKT cell population. Decreased numbers of circulating iNKT have been found in multiple tumor types such as advanced prostate cancer and are accompanied by decreased IFNγ production and increased IL-4 production by iNKT (83–85). Functional defects of iNKT have been found in human multiple myeloma where development from non-progressive or premalignant gammopathy to progressive disease was marked by a strong decrease in IFNγ producing iNKT in patient blood. However, this functional defect could be reversed by using αGalCer-pulsed matured dendritic cells (DCs) (86).

More functional iNKT defects have been described in the TRAMP prostate cancer model (TRransgenic Adenoma carcinoma of the Mouse Prostate), similar to the functional iNKT defects found in some human malignancies (87). In this model, iNKT were attracted by tumor cells to migrate into prostate tumors mediated through the CCL2-CCR5 axis. Interestingly, these primary prostate tumors as well as mouse and human prostate cancer cell lines and human prostate epithelium can express CD1d, permitting direct interaction with iNKT. Indeed, prostate tumor cells induce selective production of Th2 cytokines by iNKT and thereby bias iNKT effector functions. Interestingly, this aberrant iNKT activation was reversible by the simultaneous addition of αGalCer and IL-12, which allowed iNKT cells to produce IFNγ in response to these CD1d-expressing prostate cancer cells. Restoration of iNKT cell functions by addition of IL-12 with an agonistic CD1d ligand provides the first of several complementary novel approaches for overcoming iNKT defects in malignancy. Besides active skewing of iNKT function via interaction with CD1d on tumor cells, some tumors escape from iNKT cell lysis by loss of CD1d expression and shedding of glycolipids, such as gangliotriaosylceramide which can inhibit iNKT stimulation (88). In addition, it has been shown that iNKT can acquire suppressive functions of regulatory T cells, which is marked by nuclear expression of FoxP3 (89). Finally, an interesting example of the potential complexity of the interactions involving iNKT cells as with other immune components has been recently reported (90). Gut microbiome produced bile acids metabolites positively influenced iNKT cell accumulation and anti-tumor activity in the liver via activating liver sinusoidal endothelial cells to express iNKT chemoattractant CXCL16 (90).

As well as the protective roles for iNKT in cancer and the above-mentioned studies documenting tumor-induced alterations of iNKT cell functions, other studies have also found that some CD1d-restricted NKT cells can suppress anti-tumor responses through regulatory cytokine(s) (91, 92). These “non-invariant” NKT subsets, which are characterized by a diverse TCR repertoire are mostly referred to as type II NKT and can produce high levels of IL-13 through the IL4R/STAT6 pathway, thus promoting tumor recurrence (92) (Figure 1B). Based on these findings, Terabe et al. proposed a model in which type II NKT are responsible for downregulating tumor immunity, while type I NKT, as described above, are responsible for tumor protection (93). Additional studies reported that myeloma-derived lysophosphatidylcholine (LPC) could induce secretion of IL-13 by a small Vα24^−^Vβ11^−^ subset of NKT (94). Moreover, as IL-13 can induce production of the immunosuppressive cytokine TGF-β by MDSC (93), these data support the hypothesis of NKT driven immune suppression (Figure 1B). Together, these findings suggest a suppressive role of non-invariant NKT that could be driven by IL-13. However, our knowledge about type II NKT cells is still limited, in part due to a lack of specific markers for this subset (only CD1d tetramers with sub-optimal ligands are available) and their application is hampered by limited knowledge of glycolipid antigens specific for type II NKT. Therefore future studies are warranted to further specify the complete roles of type II NKT.

Thus far, clinical studies have mainly focused on adoptive transfer of autologous iNKT cell enriched *in vitro-*expanded populations from peripheral blood mononuclear cells (PBMCs), αGalCer-pulsed monocyte-derived DCs or a combination of activated iNKT and αGalCer pulsed DCs (95–99). Also administration of soluble αGalCer has been tested in clinical trials (98, 99). Currently, clinical benefits are still relatively limited and combinations as well as optimized strategies are being considered (96, 99). Since *ex vivo* expansion of circulating iNKT has to overcome their low frequencies in blood, induced pluripotent stem cells (iPSCs) for the generation of large numbers of iNKT might provide an alternative (100). Furthermore, a general problem with current approaches might be that although iNKT are systemically activated, their accumulation to the tumor site is not guaranteed. Targeting iNKT to the tumor microenvironment using bi-specific targeting could enhance trafficking to tumor sites and therefore increase the total anti-tumor response (101). The use of chimeric antigen receptors (CARs), which combine the targeting effect of antibodies to decrease off-target effects with the potent anti-tumor effector functions of iNKT, has been shown to be promising in pre-clinical studies and has already shown protection targeting GD2 for metastatic neuroblastoma in mice (102, 103).

As detailed above, “Type” I (invariant) NKT possess potent cytotoxic activity against cancer cells, but numerical and functional defects are limiting their full potential. Altogether, it seems that type I NKT dysfunction in cancer may be caused by acquired capacities of tumor cells to immobilize the iNKT arm of anti-tumor defense. Thereby the putative role of iNKT in immune surveillance seems to be extended toward a more controlling role in behavior of cancer cells. On the other hand, non-invariant/diverse NKT subsets (“Type II NKT”) can actively downregulate tumor immunity through different mechanisms (91–94). In the future, a more complete and evolving understanding of reversible type I NKT defects together with more insight in the mechanism behind type II NKT cell mediated suppression of antitumor immune responses (or other activities of these less understood and more diverse populations), should help the development and evaluation of novel and successful cancer therapies involving NKT populations (99, 103).

### Gamma-delta (γδ-) T cells

γδ-T cells belong to the family of unconventional T cells and differ from conventional αβ T cells, in that most γδ T cells lack expression of the CD4 and CD8 co-receptors. Intriguingly antigen recognition by the γδ TCR is not restricted to MHC- class I and II molecules (104). In humans, 0.5–16% of all CD3^+^ cells in peripheral blood and lymphoid tissues is represented by γδ T cells (105, 106). In mice, this percentage varies between 1 and 4% (107). Human γδ-T cells can be divided into two major subsets based on expression of the variable regions of TCR-δ; Vδ1, or Vδ2 (108, 109). Vδ2 cells constitute the most prominent subset in human peripheral blood and are almost always paired with Vγ9^+^ (Vγ9Vδ2) while Vδ1 are more prominent at mucosal areas (110–112). γδ T cells recognize multiple self and non-self-antigens like phospholipids, small proteins and also non-peptidic antigens, so-called pyro-phospho-antigens (pAg), either in complex with butyrophilin 3A1 (BTN3A1, CD277) or effecting a conformational change in BTN3A1/CD277 which in turn leads to Vγ9Vδ2-T cell recognition (113–116). pAgs such as (*E*)-4-hydroxy-3-methyl-but-2-enyl pyrophosphate (HMBPP) are not only produced by bacteria, but can also be produced by tumor cells with a relatively high metabolic activity of the mevalonate metabolic pathway resulting in the accumulation of pAg intermediates such as isopentenyl pyrophosphate (IPP)) (114, 117). Vγ9Vδ2 TCR mediated recognition of accumulated pAgs in tumor cells is mediated by BTN3A1/CD277 and results in strong activation and expansion of Vγ9Vδ2-T cells which is marked by the release of multiple pro-inflammatory cytokines including IFNγ, TNF-α and/or interleukin-17 (IL-17 seems to be mostly produced by Vδ1^+^ cells and it is not just a pro-inflammatory antitumor cytokine) and a strong anti-tumor response (109, 118–120) (Figure 1).

Besides activation of Vγ9Vδ2-T cells via TCR ligation, engagement of the natural killer cell receptor NKG2D contributes to the anti-tumor reactivity of Vγ9Vδ2 T and Vδ1^+^ T cells. This is especially interesting since NKG2D can bind stress- or infection induced ligands of the non-classical MHC-I related molecules H60, RAE1, and MULT in mice or MIC-A/B and ULBP1-ULBP6 in humans and while these molecules are absent on healthy cells, they are often expressed by tumor cells (121–124) (Figure 1A). Expression of ULBP molecules has been found in multiple types of cancer (leukemia, lymphoma, ovarian and colon carcinomas and hematological malignancies) and can therefore determine susceptibility to Vγ9Vδ2-T cell mediated cytolysis (125–127). Vδ1^+^ T cells not only recognize the stress induced self-antigens MICA/B via NKG2D but can also directly bind MIC molecules via their TCR (128, 129). Interestingly, enhanced expression of MICA/B by oxidative stress on tumor cells has been correlated to an increased frequency of Vδ1^+^ T cells among tumor infiltrating lymphocytes (TIL) (130).

The involvement of γδ-T cells in the elimination of tumors is at least partly based on their ability to interact with different cell types. Besides offering B cell help and triggering of DC maturation (131), Vγ9Vδ2 T cells show characteristics of antigen presenting cells, including the processing and presentation of antigens which allow the induction of naïve αβ T cell proliferation and differentiation (132, 133). This hypothesis has been further expanded by findings that Vγ9Vδ2-T cells, via trogocytosis of CD1d, can function as platform to activate iNKT in a CD1d-restricted manner (134). Since Vγ9Vδ2-T cells have the capacity to interact with different immune cells, they are important for both innate and adaptive anti-tumor responses.

The ability of γδ-T cells to generate huge amounts of pro-inflammatory cytokines, to recognize cell stress via an MHC independent mechanism, to potentiate other immune cell components, both innate and adaptive, and directly mediate cytolysis of multiple tumor types, potentially make γδ-T cells key players in anti-tumor immune responses and as such attractive therapeutic targets.

The potential impact of γδ-T cells on cancer immunotherapy has been reported in multiple studies showing γδ-T cells to be able to recognize and kill multiple different tumor types *in vitro* including leukemia, numerous carcinomas and neuroblastoma (125, 135–137). Several clinical trials have been conducted using aminobisphosphonates such as zoledronic acid (Zol) to manipulate intracellular levels of IPP (138–140). Administration of a combination of Zol with low dose IL-2 to patients with metastatic breast cancer or prostate cancer was well tolerated and increased peripheral blood Vγ9Vδ2-T cell numbers, which correlated with clinical outcome (141). In addition, synthetic pAgs, such as BrHPP have been tested in clinical trials and been shown to increase recognition of different tumor cells by Vγ9Vδ2-T cells (108). Interestingly, treatment with common chemotherapeutic compounds (e.g., temozolomide) has been shown to increase expression of stress associated NKG2D ligands on tumor cells, thereby possibly sensitizing tumor cells for Vγ9Vδ2-T recognition and opening windows for Vγ9Vδ2-T based immunotherapies (142).

While multiple studies have shown that γδ-T cells exhibit anti-tumor activity, the potential involvement of γδ-T cells in tumor progression remains rather elusive. Recently mouse and human studies emphasized a pro-tumorigenic activity of IL-17 producing and regulatory γδ-T cells (γδ T17/γδ1 Tregs) (Figure 1B). Whereas Ma et al. reported on the contribution of IL-17 producing γδ-T cells to the efficacy of anticancer chemotherapies (143), other reports showed an inverse correlation between γδ-T17 cells and overall survival, suggesting immune suppressive and tumor promoting properties of γδ-T cells by promoting accumulation of MDSCs and angiogenesis respectively (144, 145). In a transplantable model of peritoneal and ovarian cancer, γδ T17 (Vγ6^+^) cells were shown to preferentially produce IL-17 instead of IFNγ and to promote tumor growth (146). Interestingly, a reduction in tumor size was observed in TCRδ and IL-17 deficient mice compared to wild type mice, further suggesting a pivotal role of γδ T17 cells in cancer progression in this model.

Although Vγ6^+^ cells do not exist in humans, enriched amounts of Vδ1^+^ T cells with regulatory properties (γδ1-Tregs) have been identified in TIL of patients with breast cancer (128, 147). These γδ1-Tregs can suppress naïve and effector T cell responses and concordantly block maturation of dendritic cells. A more detailed in-depth study on the correlation of breast cancer TIL phenotypes with clinical outcome revealed that infiltration of γδ1-Tregs was correlated to poor prognosis (148). Together these findings imply a critical role of some γδ-T cell subsets as immune suppressors and emphasize the need for more detailed studies to better understand their regulatory functions in order to ultimately design effective innate γδ-T cell based therapeutic strategies.

Although a lot of effort has been put in understanding γδ-T cell function in tumor immunity, it is becoming clear that the overall impact of γδ-T cells in cancer treatment may depend on the fine balance between anti- and pro-tumorigenic subsets. Current challenges to optimize anti-cancer therapies lie in the quest to determine how γδ-T cell mediated anti-tumor properties can be selectively boosted, while at the same time their suppressive activity is inhibited.

### MAIT cells

Mucosal associated invariant T (MAIT) cells belong to another discrete subpopulation of T cells that is characterized by a limited TCR repertoire. Most human MAIT cells express Vα7.2-Jα33 and like in iNKT, the invariant α chain is paired with a limited diversity of TCRβ chains (Vß2 and Vß13) (149). CD161 is abundantly expressed on MAIT cells and they are highly sensitive to IL-12 and IL-18 stimulation due to their expression of IL-12R and IL-18R (150). MAIT cells recognize a variety of antigens, including bacterial and fungal derivates and metabolites of vitamin B2 (riboflavin) and B9 (folate), presented by the invariant MHC related 1 molecule (MR1) and they appear to represent important players in antimicrobial immunity (151). Their preferential location of Vα7.2-Jα33 cells is in mucosal tissue such as the gut lamina propria, but MAIT cells are also relatively abundant in peripheral blood and liver (152–154). Compared to iNKT they represent a relatively abundant cell population, with 1–4% of total TCR-αβ^+^ T cells (154). MAIT cells can secrete multiple cytokines such as IFNγ, IL-17, and TNF-α and possess lytic activity through the release of granzyme B upon activation (155, 156) (Figure 1). Since both the Th1 skewing cytokine IFNγ and the Th17 characterizing cytokine IL-17 are secreted by MAIT cells, these cells might be of great importance in the induction of either advantageous or deleterious immune responses in terms of cancer control (Figure 1).

In the past decade most publications on the roles of MAIT cells have focused on protection against infectious pathogens and in some auto immune related disorders, whereas information about their involvement in cancer immunity is relatively scarce. However, findings that accumulated MAIT cells appear to have a protective role in inflammatory bowel diseases (IBD) in humans (152) and the fact that TIL-induced intestinal inflammation present in colorectal cancer (CRC) can alter the prognosis of patients with CRC (157), suggests that intestinal MAIT cells can infiltrate into CRC tumor sites and fulfill a protective function, like in IBD. Indeed, multiple studies have recently reported active accumulation of MAIT cells in CRC while circulating activated and memory MAIT cell numbers were decreased, suggesting active homing to tumor sites (158, 159). Infiltration of MAIT cells in patients with glioblastoma and renal carcinoma in previous reports support this homing and tumor infiltrating capacity (160). Circulating MAIT cells in patients with progressive disease were significantly lower than in early stage CRC patients (158). Although it was shown that tumor infiltrating MAIT cells produced lower levels of IFNγ (and relatively high amounts of IL-17) compared to unaffected colon tissue and that this decrease was independent of lowered expression of MR1 on tumor cells (158, 161), the exact factors in the tumor microenvironment hampering antitumor effector cytokine secretion still remain elusive. Similar effects seem to apply to suppression of function of MAIT cells in CRC metastases to the liver (162). Finally, such defects may be common to a wide variety of cancers (163), since their numbers and activity are also reduced in myeloma patients, although which came first: the defects or the cancer, was a question raised by the finding that carefully age matched (generally older) people have reduced MAIT cells (164).

Until now, little is known about MR1 distribution and it has yet to be elucidated whether MR1 expression on tumor cells could be important in MAIT cell activation. Furthermore, in order to better understand MAIT cell interactions in neoplasms and to exploit MAIT cells for immune therapies, more detailed studies on new ligands and ligand driven expansion are urgently needed.

## Conclusions: combining innate immune therapies with chemo- and other therapies

Innate immune cells may represent the first line of defense against malignancies, e.g., through the MHC-independent recognition of their metabolically stressed state, and their potency as regulators and mediators of tumor immune responses, both innate and adaptive, has been widely acknowledged as discussed above (Figure 1). Therefore classical NK cells and the different innate (semi)-invariant T cell subsets have garnered interest in the field of anti-cancer immunotherapies and multiple NK, iNKT, and γδ-T based immunotherapeutic approaches are currently clinically tested. Although these therapies show some promising results, overall clinical benefits are still limited and the explored strategies need to be optimized. Identification of mechanisms underlying NK, iNKT, γδ-T, and MAIT cells defects, which have been observed in patients with cancer, could fuel the development of alternative approaches to current treatments. For example, in the case of immune editing to evade NK effector function, blockade of inhibitory receptors on NK cells to overcome NK paralysis has made its first steps in clinical trials. Overall, defined molecular mechanisms and interactions of tumor cells with immune cells in the tumor microenvironment need to be further investigated in order to understand how local immune suppression of effector cells can be overcome.

There is a general consensus that in order for immunotherapy to be fully effective combinatorial therapies need to be developed and clinically tested. Multiple studies have indicated that low-dose chemotherapeutics can reduce local immune suppression by, for example elimination of MDSC in the tumor microenvironment (165–167), and therefore increase efficacy of already applied immune therapies. Since IL-13 producing type II NKT have been associated with immune suppression mediated via MDSC-derived TGF-β, combining NKT based therapies with chemotherapeutics might reverse the flavor of NKT in this case from “bad” to “good” in terms of cancer control. Therefore, combining immune therapy with chemo-therapy could perhaps also benefit innate immunity based anti-tumor therapies in certain circumstances. Moreover, the immune system comprises many elements which are tightly regulated and connected and as such cross-talk of innate effector cells with each other or with other immune cells could be exploited to enhance the efficacy of current therapies. For example, close crosstalk between iNKTs and Vγ9Vδ2-T cells has been described, in such a way that αGalCer activated iNKT to enhance CD25 expression and IFNγ production of γδ-T cells via secretion of TNF-α (168). The findings that iNKT can potentiate antitumor effector functions of Vγ9Vδ2-T cells and that Vγ9Vδ2-T cells possess unique features to activate iNKT, opens up new avenues to strengthen future iNKT and Vγ9Vδ2 T cell based immunotherapeutic approaches. An alternative approach for combinatorial therapy could be to enhance the interaction of iNKT with DCs using vehicles/vaccines to target both types of cells in order to maximize anti-tumor effects. Indeed, an OVA peptide/CpG vaccine combined with recombinant α-galactosylceramide (αGC)-loaded CD1d-anti-HER2 fusion protein showed increased expansion of OVA-specific CTLs and was likely mediated via maturation of DCs (169).

Nowadays, great advances have been made in the available detection methods for monitoring immune cells in tumors using mass cytometry. Also next generation (single cell) sequencing of (invariant) T cells has proven itself to be helpful for the identification of new invariant T cells (170). These big data approaches facilitate identification and detailed analysis of immune cells and their plasticity in malignancies and will hopefully contribute to a better understanding of dualistic roles of innate cells in cancer control and progression.

In conclusion, innate immune effector (NK/T) lymphocyte subsets are key in regulating cancer control versus progression. If present hurdles can be overcome and the fine line between their suppression or progression of tumor growth has been further elucidated, NK cells, iNKT, γδ T, and MAIT cells hold great promise for the induction of long lasting anti-tumor immunity.

## Author contributions

ME received and accepted the commission, recruited the coauthors, and edited the manuscript. TG, YK and HV edited the manuscript. DS drafted and edited the manuscript and drew the figure.

### Conflict of interest statement

The authors declare that the research was conducted in the absence of any commercial or financial relationships that could be construed as a potential conflict of interest.
